# Endocytosis of Albumin Induces Matrix Metalloproteinase-9 by Activating the ERK Signaling Pathway in Renal Tubule Epithelial Cells

**DOI:** 10.3390/ijms18081758

**Published:** 2017-08-12

**Authors:** Xiaoming Chen, Alyssa Cobbs, Jasmine George, Ashmeer Chima, Fidele Tuyishime, Xueying Zhao

**Affiliations:** Department of Physiology, Morehouse School of Medicine, Atlanta, GA 30310, USA; xachen@msm.edu (X.C.); acobbs@msm.edu (A.C.); Jgeorge@msm.edu (J.G.); achima@msm.edu (A.C.); ftuyishime@msm.edu (F.T.)

**Keywords:** chronic kidney disease, diabetes, MMP-9, albuminuria, U0126, simvastatin

## Abstract

Matrix metalloproteinase-9 (MMP-9) is dysregulated in chronic kidney diseases including diabetic nephropathy. This study was performed to examine the expression of MMP-9 in renal tubule epithelial cells (TECs) under diabetic conditions and its regulatory mechanisms. We characterized MMP-9 protein in diabetic animals and primary cultured rat TECs exposed to exogenous albumin and high glucose. We also used specific inhibitors to determine if internalization of albumin and/or extracellular signal-regulated kinase 1/2 (ERK1/2) phosphorylation were required for MMP-9 secretion. Immunostaining of kidney sections revealed enhanced MMP-9 signal in the damaged proximal tubules in Zucker diabetic fatty (ZDF) rats. ZDF rats also exhibited an albuminuria-related and age-dependent increase in MMP-9 excretion, which was prevented by rosiglitazone. In primary cultured rat TECs, high glucose exposure did not increase MMP-9 secretion. In contrast, administration of rat serum albumin (RSA, 0.1–0.5 mg/mL) resulted in a dose-dependent increase in MMP-9 expression and secretion by TECs, which was abolished in the presence of an ERK1/2-specific inhibitor, U0126. Simvastatin, an inhibitor of albumin endocytosis, also prevented MMP-9 secretion. Taken together, these results demonstrate that endocytosis of albumin stimulates MMP-9 secretion by TECs through the ERK signaling pathway.

## 1. Introduction

Diabetic nephropathy (DN) is now the leading cause of end-stage renal disease and accounts for over one-third of all patients who are on dialysis. DN is characterized by early albuminuria, which often progresses to proteinuria and ultimately leads to a decline in excretory renal function associated with glomerulosclerosis and tubulointerstitial fibrosis [1,2]. Albumin has been shown in proximal tubule epithelial cells (TECs) to induce the production of a variety of proinflammatory and profibrotic cytokines such as regulated on activation, normal T cell expressed and secreted (RANTES) [3], monocyte chemoattractant protein-1 (MCP-1) [4,5], and transforming growth factor-β (TGF-β) [6,7], which involves both endocytosis-dependent and -independent mechanisms.

Matrix metalloproteinases (MMPs) were previously known to be anti-fibrotic for their ability to degrade and remodel the extracellular matrix (ECM). Recent studies have shown that MMPs, especially gelatinases (MMP-2 and MMP-9), are involved in the initiation and progression of kidney fibrosis [8]. An increase in MMP-2 and MMP-9 is associated with the induction of tubular cell epithelial–mesenchymal transition (EMT) in vitro [9] and in vivo [10]. We, and others, have previously demonstrated an increase in urinary MMP-9 excretion in animal models of diabetic kidney disease and proteinuric human disease [11,12,13,14,15,16,17]. Since albumin filters through the glomerulus into the tubular lumen and is reabsorbed by proximal tubules [18], we hypothesized that albumin overload would enhance the expression and secretion of MMP-9 by renal TECs.

To this end, we first performed immunofluorescence staining to evaluate albumin reabsorption and MMP-9 protein expression in the proximal tubules of Zucker lean (ZL) and Zucker diabetic fatty (ZDF) rats. Apart from regulating hyperglycemia, thiazolidinediones (rosiglitazone, pioglitazone and troglitazone) have been shown to be renoprotective by reducing albuminuria and proteinuria in experimental animals and patients with diabetes [19,20,21,22]. Thus, MMP-9 excretion was also analyzed in ZDF rats treated with rosiglitazone. Next, we examined the direct effect of high glucose and albumin on the expression and secretion of MMP-9 by primary cultured rat TECs following exposure to high glucose or rat serum albumin (RSA). The roles of ERK1/2 activation and albumin endocytosis in the production of MMP-9 by TECs were assessed in the absence and presence of specific inhibitors.

## 2. Results

### 2.1. Tubular Expression and Urinary Excretion of MMP-9 Protein Were Increased in Association with Albumin Overload in the Diabetic Kidneys

**Figure 1:**
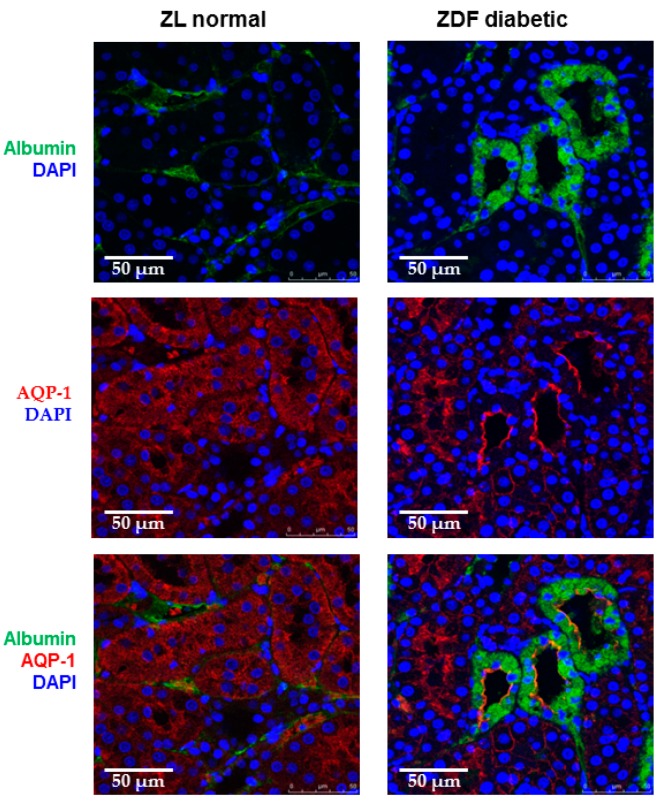
Albumin aggregation in proximal tubule epithelial cells of diabetic kidneys. Dual labeling using antibodies specific for rat albumin (green) and aquaporin-1 (AQP-1, red) reveals an accumulation of albumin in the AQP-1-positive tubules of 20-week-old Zucker diabetic fatty (ZDF) rats compared to Zucker lean (ZL) normal controls. Cell nuclei were stained by DAPI (blue).

We first confirmed an increase in the urinary protein-to-creatinine ratio in 20-week-old ZDF rats (36 ± 3, *n* = 7, *p* < 0.01) compared to ZL normal controls (10 ± 2, *n* = 8). To evaluate the reabsorption of endogenous albumin by proximal tubules, kidney sections of ZL and ZDF rats were stained with anti-rat albumin and anti-aquaporin-1 antibodies. We found that a subset of ZDF tubules stained intensely for albumin, whereas albumin was barely present in the tubules of age-matched ZL controls (Figure 1). Intense albumin staining was predominantly detected in aquaportin-1-positive tubules, suggesting an accumulation of albumin in proximal TECs of ZDF kidneys. Moreover, positively stained ZDF tubules were often dilated and appeared as clusters.

**Figure 2:**
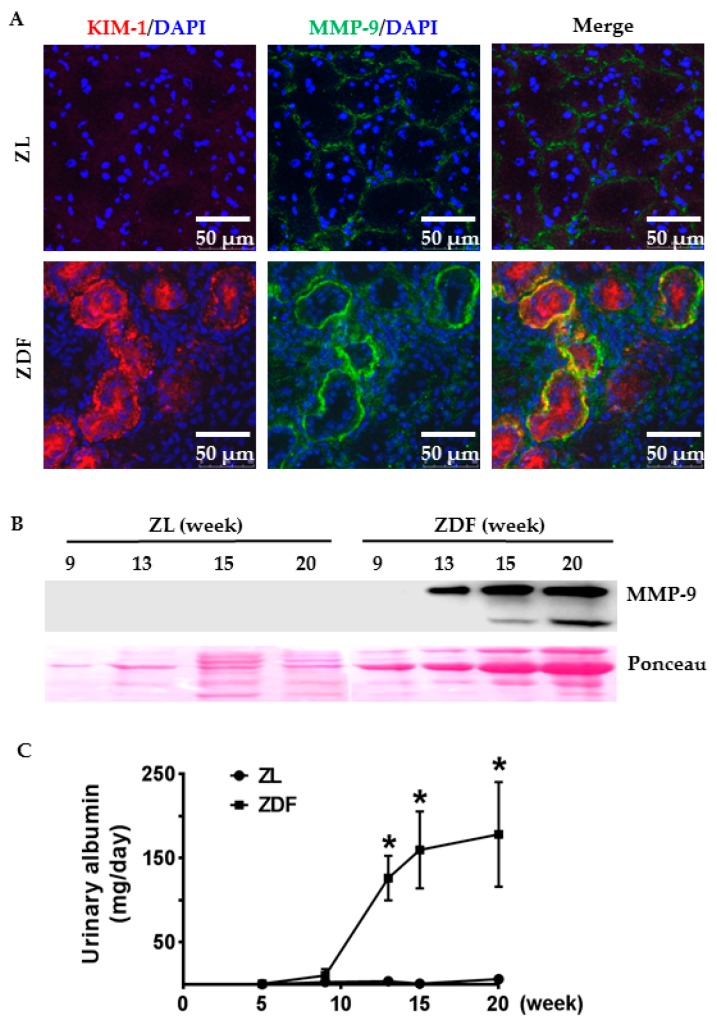
Increased tubular expression and urinary excretion of MMP-9 protein in ZDF rats. (**A**) Cell nuclei were stained by DAPI (blue). Double immunofluorescence staining shows that MMP-9 (green) signal was enhanced on the basolateral membrane of damaged tubules with strong kidney injury molecule-1 (KIM-1) (red) staining in 20-week-old ZDF rats. As expected, KIM-1 staining was absent in normal ZL kidneys. Weak linear MMP-9 staining was detected along normal tubules; (**B**) Representative Western blotting images of MMP-9 and Ponceau red staining show a gradual increase in MMP-9 protein in the urine collected from nine- to 20-week-old ZDF rats, whereas MMP-9 was not detectable in the urine of ZL rats; (**C**) Urinary albumin levels were determined in 24-h urine collected from five- to 20-week-old ZL and ZDF rats. Values are mean ± SEM; *n* = 4–7; * *p* < 0.01 vs. age-matched ZL controls.

To examine the expression pattern of MMP-9 in diabetic rat tubules, we performed dual labeling for MMP-9 and kidney injury molecule-1 (KIM-1), a proximal tubule injury marker. As expected, KIM-1 staining was negligible in ZL kidneys (Figure 2A: ZL). ZDF rats exhibited a heterogeneous staining pattern with intense KIM-1 signal in clusters of ZDF tubules (Figure 2A: ZDF). Compared to the weak MMP-9 signal along normal tubules of ZL rats (Figure 2A: ZL), MMP-9 staining was enhanced in KIM-1-positive ZDF tubules (Figure 2A: ZDF). A co-localization of MMP-9 and KIM-1 was detected on the basolateral membrane of proximal tubules, though KIM-1 staining was present in both apical and basolateral membranes.

To dynamically monitor MMP-9 induction in the diabetic kidneys, we quantified MMP-9 protein levels in the urine samples of nine- to 20-week-old Zucker rats. As shown in Figure 2B, MMP-9 was not detected by Western blotting in normal ZL rat urine at any age. In contrast, ZDF rats demonstrated an age-dependent increase in urinary MMP-9. MMP-9 protein in ZDF urine was barely detectable at nine weeks but became abundant at 13 weeks and further increased at 15 and 20 weeks (Figure 2B). This increase in MMP-9 excretion was closely correlated to the level of urinary albumin in ZDF rats (Figure 2C).

### 2.2. Rosiglitazone Reduced Blood Glucose and Urinary Excretion of Albumin and MMP-9 in ZDF Rats

**Figure 3:**
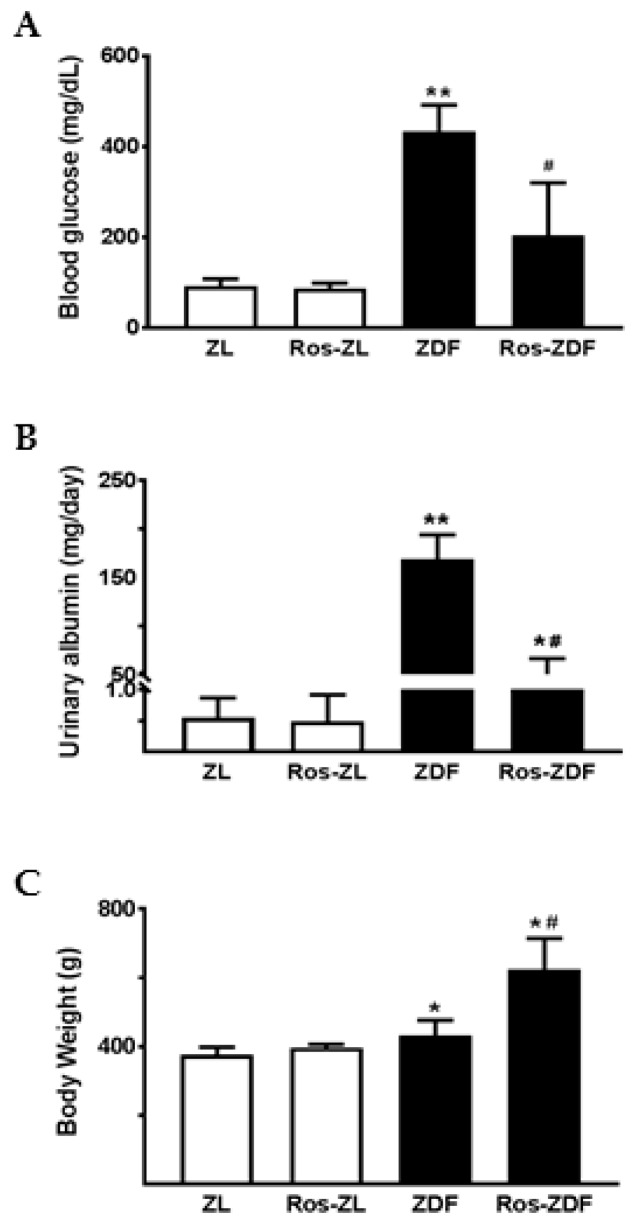
Effect of rosiglitazone on blood glucose, urinary albumin, and body weight in Zucker rats. Rosiglitazone reduced blood glucose (**A**) and urinary albumin (**B**) levels but increased body weight (**C**) in ZDF rats. Values are mean ± SEM. *n* = 4–7 rats; * *p* < 0.05 and ** *p* < 0.01 compared with their ZL controls (ZDF vs. ZL or Ros-ZDF vs. Ros-ZL); ^#^ *p* < 0.05 compared to vehicle-treated ZDF group (Ros-ZDF vs. ZDF).

**Figure 4:**
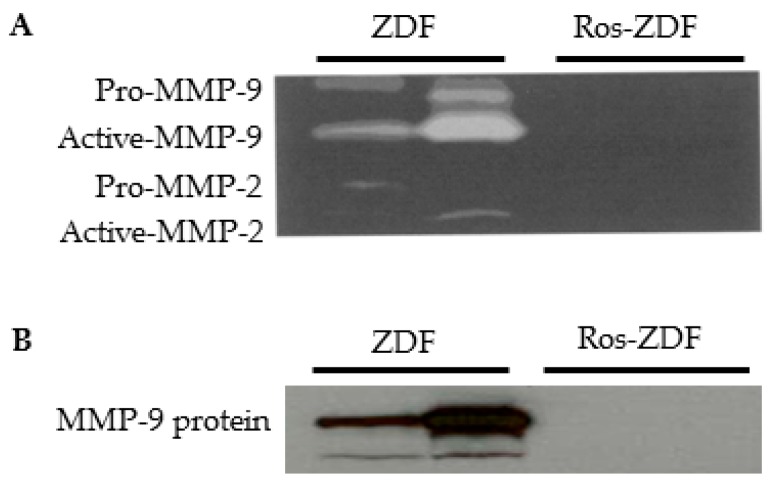
Effect of rosiglitazone on urinary gelatinolytic activity and MMP-9 protein in ZDF rats. (**A**) Representative gelatin zymograph shows a decrease in urinary MMP-9 and MMP-2 activity in ZDF rats following rosiglitazone treatment; (**B**) Western blot confirms a decrease in urinary MMP-9 protein in rosiglitazone-treated ZDF rats compared to vehicle-treated ZDF ones.

To examine the effect of PPARγ activation on MMP-9 excretion, 12-week-old ZL and ZDF rats were treated with rosiglitazone for eight weeks. Metabolic effects of rosiglitazone are shown in Table 1 and Figure 3. In ZL rats, blood glucose, urinary albumin, and body weight remained normal following rosiglitazone administration (Figure 3A–C). Gelatinolytic activity and MMP-9 protein were not detected by gelatin zymography and Western blotting in both vehicle and rosiglitazone-treated ZL rat urine. As expected, chronic treatment of ZDF rats with rosiglitazone significantly reduced blood glucose and urinary excretion of protein and albumin but increased body weight. As depicted in Figure 4, rosiglitazone treatment resulted in a suppression of MMP-9 excretion in ZDF rats. MMP-2 activity was also reduced in rosiglitazone-treated ZDF rat urine.

### 2.3. Primary Cell Culture and Albumin Endocytosis

**Figure 5:**
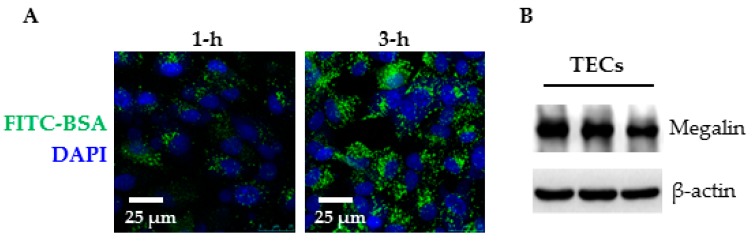
Albumin endocytosis and megalin expression in primary rat TEC. Rat TECs were incubated with 100 µg/mL FITC-BSA at 37 °C for 1 h (**A**: 1-h) or 3 h (**A**: 3-h) and then washed and fixed. Fluorescence was visualized by confocal microscopy. Representative Western blot images show megalin protein expression in primary TECs (**B**).

Isolated tubular fragments from rat kidneys were used for cell culture. After 6–7 days, tubular outgrowth became progressively larger and formed a monolayer with polygonal morphology and homogeneous appearance. To further characterize the cells, albumin endocytosis and immunoblotting for megalin, a major component of the receptor-mediated endocytosis, were performed. To directly visualize the endocytic uptake of albumin by primary TECs, the cells were incubated with FITC-conjugated bovine serum albumin (BSA) for 1–3 h. Representative confocal images show FITC-BSA staining in vesicular punctuate structures after one-hour incubation (Figure 5A). Small albumin-containing vesicles aggregated to form larger structures after three-hour incubation. Moreover, megalin was clearly detected in the cell lysates of TECs (Figure 5B).

### 2.4. Albumin Exposure Induced MMP-9 Expression and Secretion by Primary Rat TECs

**Figure 6:**
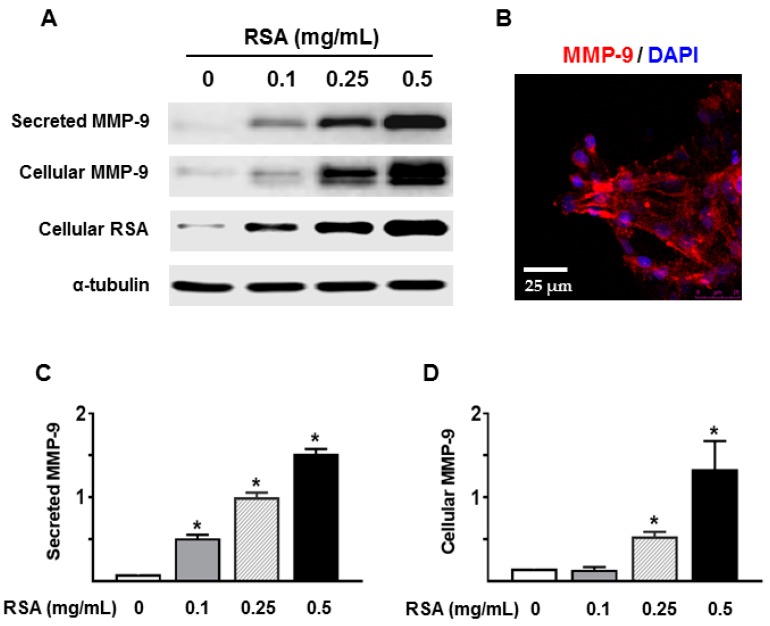
Albumin increased MMP-9 protein expression and secretion by primary rat TECs. Rat serum albumin (RSA 0.1–0.5 mg/mL) administration for 48 h resulted in a dose-dependent increase in MMP-9 protein in culture supernatants (**A**,**C**) and cell homogenates (**A**,**D**) of primary cultured rat TECs; (**B**) Representative confocal image shows MMP-9 signal is predominantly present on the cell surface of TECs. Values are mean ± SEM. *n* = 4; * *p* < 0.05 vs. untreated control group.

We next determined whether albumin overload directly stimulated MMP-9 expression and secretion by renal TECs. Primary cultured TECs were incubated with different concentrations of RSA (0.1–0.5 mg/mL), which is mainly globulin-free. RSA incubation of TECs resulted in a dose-dependent increase in MMP-9 expression and secretion (Figure 6). Immunofluorescence labeling revealed that MMP-9 signal was primarily present on the cell surface of cultured TECs (Figure 6B).

**Figure 7:**
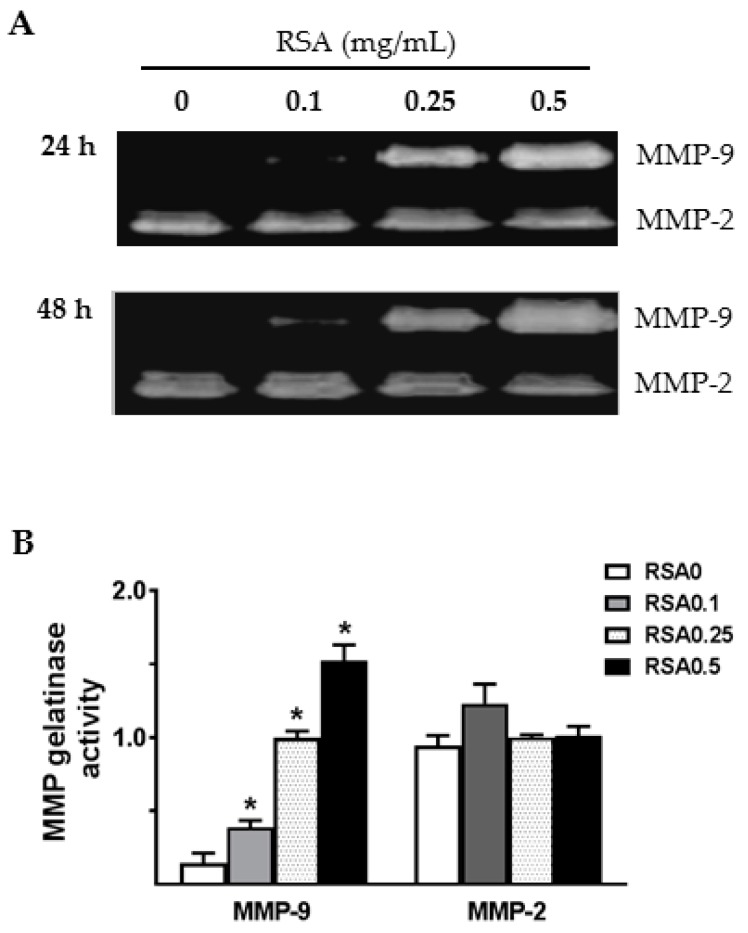
MMP-9 activity was increased in culture supernatants of primary rat TECs upon albumin stimulation. Gelatin zymography analysis confirms a dose-dependent increase in MMP-9 activity in the culture supernatants when the cells were incubated with rat serum albumin (RSA, 0.1–0.5 mg/mL) for 24 h (**A**) or 48 h (**A**,**B**), whereas MMP-2 activity was not altered. Values are mean ± SEM. *n* = 4; * *p* < 0.05 vs. untreated control group.

MMP-2 and MMP-9 gelatinolytic activities were further evaluated in the conditioned media of TECs by gelatin zymography. As shown in Figure 7, a dose-dependent increase in the gelatinolytic activity of MMP-9 was observed following RSA treatment. To investigate whether the increase in MMP-9 was specific to the type of albumin (globulin-free or fatty acid-free RSA), primary TECs were treated with the same concentration of either the globulin-free RSA used above or the fatty acid-free RSA (FA-RSA). FA-RSA induced an increase in MMP-9 that was similar to that produced by globulin-free RSA. Compared to a low level of MMP-9 activity in culture supernatants of untreated TECs, the basal level of MMP-2-related gelatinase activity was relatively high (Figure 7). However, none of the albumin preparations tested above induced a change in MMP-2 release from cultured TECs.

### 2.5. High Glucose Alone Did not Induce MMP-9 Secretion by Primary Rat TECs

**Figure 8:**
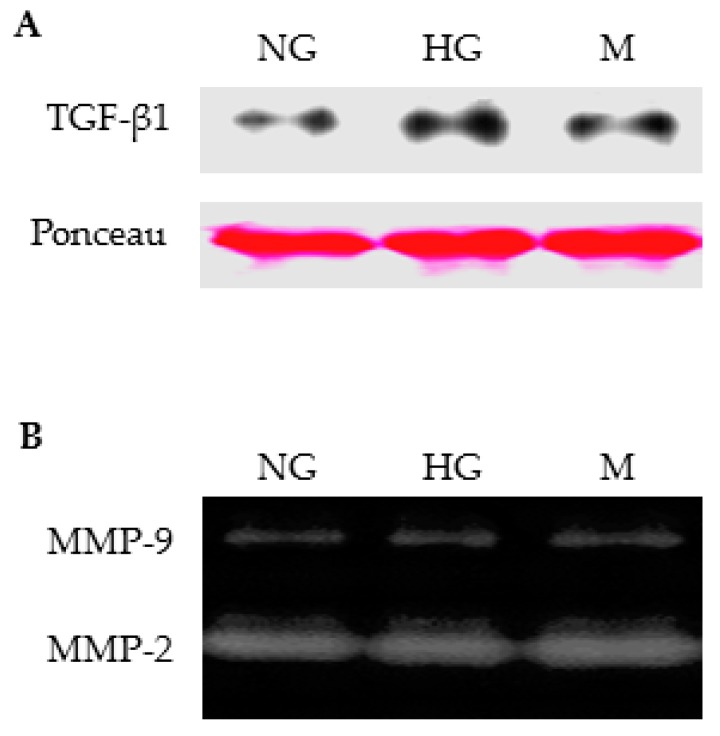
Effect of high glucose on secretion TGF-β1 and MMP-9 by primary rat TECs. (**A**) Representative Western blotting images show TGF-β1 protein in culture supernatants of TECs treated with normal glucose (NG), high glucose (HG) and mannitol (M) for 24 h. Ponceau red staining was used as loading control; (**B**) Gelatin zymograph shows MMP-2 and MMP-9 activities in culture supernatants of TECs treated with normal glucose, high glucose, or mannitol for 24 h.

We also evaluated the effect of high glucose on secretion of gelatinases by cultured TECs. Western blotting analysis showed an increase in TGF-β1 secretion when TECs were incubated with high glucose for 24 h (Figure 8A). However, high glucose exposure did not induce MMP-9 secretion by TECs. MMP-9 activities in culture supernatants remained unchanged in TECs treated with high glucose for 24 h (Figure 8B) or 48 h (data not shown). Similarly, MMP-2 secretion was not affected by high glucose treatment (Figure 8B).

### 2.6. Albumin-Induced MMP-9 Was Dependent on ERK1/2 Activation in Cultured TECs

**Figure 9:**
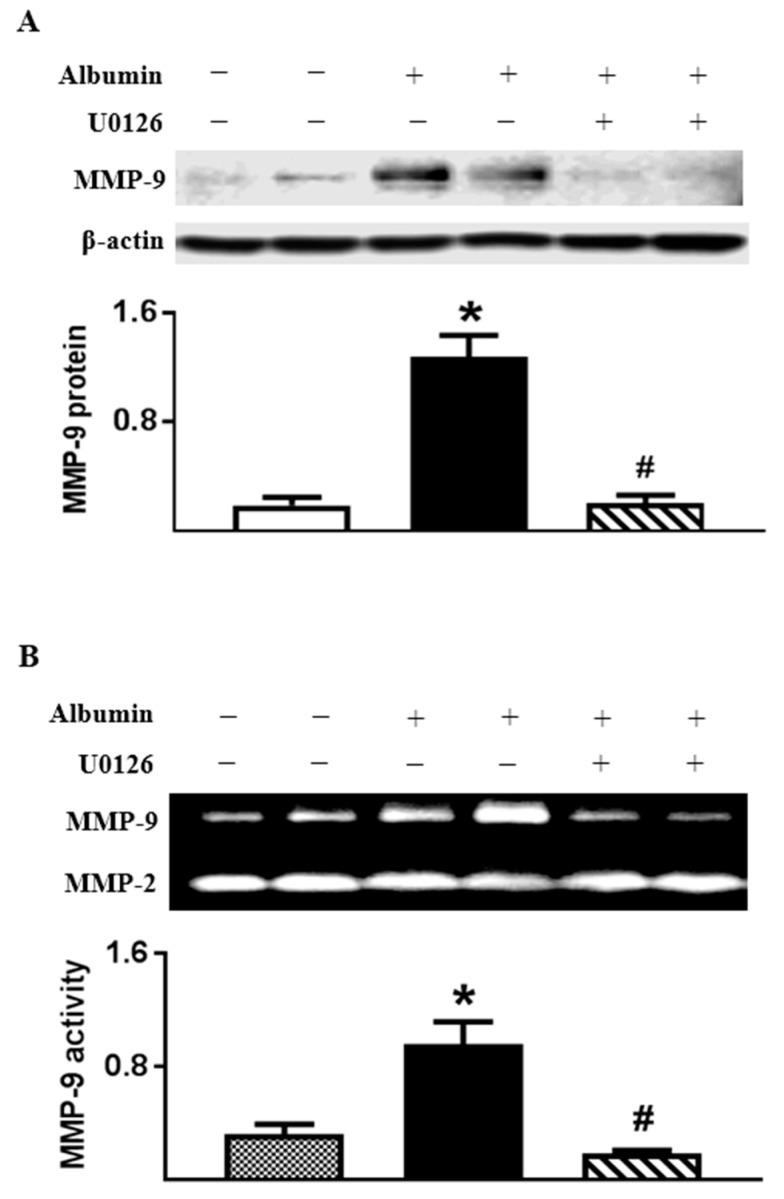
Albumin-induced MMP-9 was inhibited by U0126 in primary rat TECs. The cells were exposed to RSA (0.5 mg/mL) for 24 h in the absence or presence of U0126. (**A**) U0126 abolished albumin-induced MMP-9 protein in TECs; (**B**) MMP-9 activity was increased in culture supernatant of RSA-treated TECs, which was suppressed in the presence of U0126. Values are mean ± SEM. *n* = 4; * *p* < 0.05 vs. unstimulated normal control; ^#^ *p* < 0.05 vs. vehicle-treated albumin-stimulated cells.

We next sought to understand the mechanism for albumin-induced MMP-9 production in TECs. Our recent study [16] indicated that ERK1/2 activation was required for MMP-9 induction in glomerular epithelial cells upon albumin stimulation. Here, we show that ERK1/2 phosphorylation was increased in RSA-treated TECs (phospho-ERK1/2 to total ERK1/2 ratio: 0.53 ± 0.05, *n* = 4, *p* < 0.05) compared with vehicle control (0.29 ± 0.01, *n* = 4). In addition, albumin-induced MMP-9 expression and secretion by TECs were suppressed by U0126, an inhibitor of the ERK1/2 signaling pathway. In the presence of U0126, MMP-9 protein in cell lysates and activity in culture supernatants were normalized to basal levels in RSA (0.5 mg/mL)-treated TECs (Figure 9). However, inhibition of ERK1/2 activation did not alter MMP-2 secretion by RSA-treated TECs.

### 2.7. Inhibiting Endocytosis Attenuated Albumin-Induced MMP-9 Secretion by TECs

**Figure 10:**
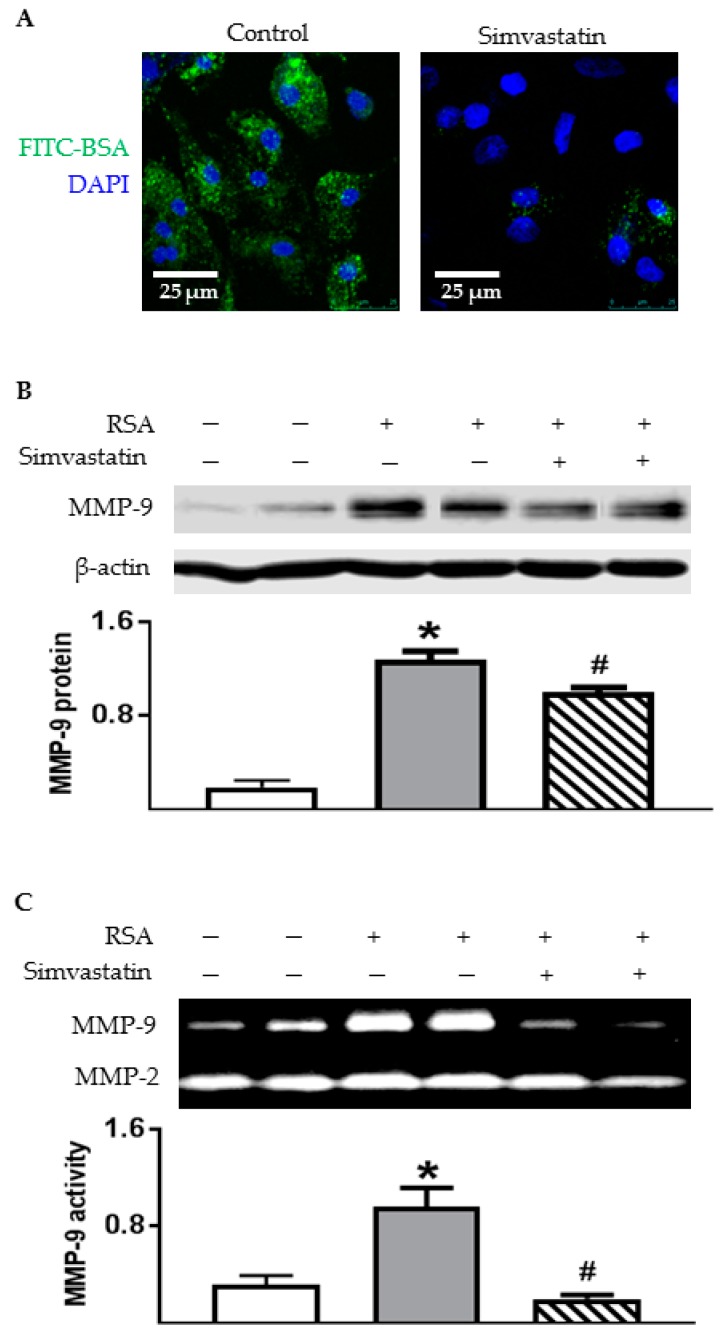
Albumin-induced MMP-9 production was attenuated by simvastatin treatment in primary rat TECs. (**A**) Representative confocal images show decreased FITC-BSA signal in simvastatin-treated TECs compared to DMSO vehicle controls; (**B**) Western blot analysis of MMP-9 protein in TECs treated with RSA (0.5 mg/mL) for 24 h in the absence or presence of simvastatin (10 µM); (**C**) Gelatin zymograph of MMP-2 and MMP-9 activities in the culture supernatants of TECs. Values are mean ± SEM, *n* = 4; * *p* < 0.05 vs. unstimulated normal control; ^#^ *p* < 0.05 vs. vehicle-pretreated albumin-stimulated cells.

To investigate the role of endocytosis in albumin-induced MMP-9 production, the TECs were pretreated with simvastatin and then exposed to RSA. Simvastatin (10 µM), a 3-hydroxy-3-methylglutaryl CoA reductase inhibitor, has been shown to reduce albumin endocytosis by about 45% in opossum kidney cells [6,23,24]. Using confocal microscopy, we confirmed a reduction of FITC-BSA uptake by TECs in the presence of simvastatin (Figure 10A). As depicted in Figure 10B, simvastatin significantly attenuated the upregulation of MMP-9 protein in TECs. Accordingly, albumin-induced MMP-9 secretion was prevented by simvastatin pretreatment (Figure 10C). Nevertheless, inhibition of endocytosis did not affect MMP-2 secretion from cultured TECs.

## 3. Discussion

Dysregulation of renal MMP-9 production has been reported in several different types of proteinuric kidney diseases including diabetic nephropathy [11,12,13,14,15,17,25,26]. In this study, we have expanded these findings by showing that (1) albumin overload was associated with increased MMP-9 secretion by damaged proximal TECs in ZDF rats; (2) rosiglitazone inhibited albuminuria-associated MMP-9 excretion in ZDF rat urine; (3) internalization of albumin stimulated MMP-9 secretion by primary cultures of rat TECs via the activation of ERK1/2 signaling pathway.

We first confirmed an accumulation of albumin in the proximal TECs of ZDF rats. Albumin filters through the glomerulus into the tubular lumen and is reabsorbed by the megalin/cubilin complex on the apical membrane of proximal tubules [18]. Growing evidence supports the idea that the tubulointerstitial nephrotoxic effects of proteinuria are dependent on the tubular uptake of proteins. However, a reduction in the reabsorption efficiency of albumin in the diabetic rats has been suggested since less filtered fluorescent albumin was detected in the renal proximal tubule cells of diabetic rats [27,28]. In those studies, a fixed amount of labeled albumin was injected to control and diabetic rats, which allowed for the estimation of the reabsorption ratio or efficiency by quantitating fluorescence of exogenous protein, but not absolute reabsorption amount of endogenous albumin. Using immunostaining for rat albumin, we detected an increase in albumin staining in aquaporin-1-positive cells of ZDF rat tubules, suggesting that albumin overload occurred in the proximal tubules of diabetic rats. This finding is consistent with a heterogeneous pattern of intense albumin staining described by Kralik et al. [29] in diabetic OVE26 mouse and proteinuric human kidneys. Moreover, Mori et al. found that reabsorption of endogenous albumin was doubled in STZ-induced or Akita-inherited type 1 diabetic mice and reabsorption efficiency was reduced at the same time [30]. Protein accumulation may occur because of an increase in reabsorption or an insufficient ability to process and dispose of internalized protein by proximal tubule cells [31]. Further studies are warranted to determine whether albumin accumulation in diabetic rat tubules is due to enhanced albumin uptake or a failure to clear internalized albumin.

MMP-9 activation is closely linked to albuminuria/proteinuria in animal models of proteinuric kidney diseases and in human nephropathies [11,12,13,14,15,17]. Our results provide evidence for an albuminuria-related and age-dependent increase in MMP-9 excretion in ZDF rats. Dual labeling for MMP-9 and KIM-1 revealed that enhanced MMP-9 signaling was predominantly present on the basolateral membrane of damaged proximal tubules in ZDF rats. Given its role in degradation of tubular basement membrane, macrophage recruitment via osteopontin cleavage and the development of tubulointerstitial fibrosis in proteinuric kidney diseases [10,32], MMP-9 activation on the basolateral membrane of renal tubules in diabetic kidney is functionally important. Local secretion of MMP-9 may play an important role in nephropathy progression by facilitating the degradation of the tubular basement membrane, resulting in an inflammatory and fibrotic response in the interstitium of diabetic kidneys.

In the current study, we also confirmed a renoprotective effect of thiazolidinediones by showing that albuminuria and proteinuria were attenuated in rosiglitazone-treated ZDF rats. The diabetic rats demonstrated weight gain following rosiglitazone administration for 8 weeks. This increase in body weight can be caused by fluid retention, one of well-documented side effects of thiazolidinediones [19,21]. The reduction of blood glucose and urinary albumin was associated with a decrease in MMP-9 excretion in rosiglitazone-treated ZDF rats. These in vivo results suggest a possible stimulatory role for high glucose and/or albumin in renal MMP-9 production. However, incubation of TECs with high glucose alone did not induce MMP-9 secretion despite an increase in TGF-β1 secretion. This is in agreement with our previous finding that high glucose or TGF-β1 stimulation does not increase MMP-9 secretion from glomerular epithelial cells [16].

Next, we tested the hypothesis that albumin overload or increased protein in the lumen could trigger tubular expression and secretion of MMP-9 in primary cultured rat TECs. A significant elevation of MMP-9 activity was detected in culture supernatants when the cells were exposed to RSA. Albumin-induced MMP-9 secretion seems to be independent of the fatty acids bound to albumin since there is no significant difference in MMP-9 secretion by globulin-free and fatty acid-free RSA-treated TECs. Future studies should consider whether and how RSA stimulation induces pro-inflammatory and pro-fibrotic mediators in primary rat TECs.

There are different cellular mechanisms involved in albumin-induced pro-inflammatory and pro-fibrotic cytokines in proximal tubules. Some of the responses of proximal TECs to albumin are known to be dependent on albumin endocytosis such as MCP-1 [5]. In contrast, albumin-induced TGF-β1 secretion is independent of endocytosis [6]. Hence, the current study also assessed the role of albumin endocytosis in the secretion of MMP-9 by TECs in the presence of simvastatin. Simvastatin has been shown to significantly inhibited the uptake of protein by opossum kidney (OK) cells [6,23] and human proximal tubule cells [24] as a result of inhibition of HMG–CoA reductase. We confirmed an inhibitory effect of simvastatin on albumin uptake by TECs. Moreover, albumin-induced MMP-9 expression and secretion were inhibited by simvastatin, suggesting that internalization of albumin is required for the activation of MMP-9 in rat TECs.

The MAPK signaling cascade is known to mediate the activation of NFκB and NFκB-dependent genes such as IL-8 [33] and MCP-1 [4,5] in proximal TECs upon albumin stimulation. To determine if ERK1/2 phosphorylation is required for albumin-induced MMP-9 secretion, primary TECs were exposed to RSA in the presence of U0126. Inhibiting ERK1/2 phosphorylation in TECs using U0126 had no effect on albumin endocytosis (data not shown) but significantly reduced MMP-9 protein expression and secretion. These observations support the role of ERK1/2 signaling pathway in albumin-induced MMP-9 production by TECs.

## 4. Materials and Methods

### 4.1. Experimental Animals

Male ZL and ZDF rats were purchased from Charles River Laboratories (Wilmington, MA, USA). Rats were housed in a temperature-controlled room with a 12:12-h light–dark cycle and free access to Purina 5008 rat chow and water. Fasting blood glucose was measured once a week after a 6-h fast. Urine was collected over a 24-h period every two weeks in metabolic cages and stored at −80 °C. In one set of experiments, 12-week-old ZDF rats were treated with vehicle (5% cyclodextrin in drinking water) or rosiglitazone (Ros, 10 mg/kg/day in drinking water) for eight weeks. All experimental procedures were performed in accordance with the National Institutes of Health Guide for the Care and Use Laboratory Animals and approved (approval number 15-22) on 30 November 2015 by the Morehouse School of Medicine Animal Care and Use Committee.

### 4.2. Measurement of Urinary Albumin, Protein, and Creatinine

Urinary albumin was measured using a commercial Nephrat kit (Exocell, Philadelphia, PA, USA), and total protein was determined via the Bradford method (Bio-Rad Protein Assay kit, Bio-Rad Laboratories, Hercules, CA, USA). A modified kinetic Jaffe reaction was performed to determine the urine creatinine level. The ratio of urine protein to creatinine was calculated by dividing the urine protein concentration by the creatinine concentration, both expressed in mg/dL.

### 4.3. Culture of Primary Tubular Epithelial Cells

Six- to ten-week-old Sprague–Dawley rat kidneys were dissected by a modified procedure as described previously [34]. The tubular fragments were collected and cultured in growth medium containing F12/DMEM (5 mM d-Glucose), 10% fetal bovine serum, penicillin, and streptomycin (Life Technologies, Carlsbad, CA, USA) until TECs reached 80–90% confluency (day 6–7). The cells were washed with serum-free DMEM and treated with globulin-free or fatty acid-free rat serum albumin (RSA, Sigma Aldrich Inc., St. Louis, MO, USA) at varying concentrations (0, 0.1, 0.25, or 0.5 mg/mL) prepared in 2% FBS/DMEM/F12 medium for 24–48 h. Culture supernatants were collected, and cell lysates were prepared using RIPA lysis buffer with a cocktail of protease inhibitors (Sigma Aldrich Inc.). To determine the effect of albumin on ERK1/2 activation, the cells were treated with RSA (0.25 mg/mL) for 90 min, and cellular protein was extracted using PhosphoSafe extraction reagent (MED Millipore, Temecula, CA, USA). In another set of experiments, the effect of high glucose on MMP-9 production was evaluated by incubating the TECs with 2% FBS/DMEM/F12 containing either 5 or 30 mM d-glucose medium for 24–48 h. The effect of hyperosmolality was assessed in TECs cultured in DMEM/F-12 containing 5 mM d-glucose supplemented with 25 mM mannitol.

### 4.4. Effect of Inhibitors on MMP-9 Release

To evaluate the role of ERK1/2 MAP kinases in albumin-induced MMP-9 release, the TECs were exposed to RSA (0.5 mg/mL) in the presence of U0126 (1,4-diamino-2,3-dicyano-1,4-bis (2-amino phenylthio)) butadiene (Sigma Aldrich Inc.), a selective ERK1/2 inhibitor. Additionally, simvastatin (Sigma Aldrich Inc.) was used to inhibit albumin endocytosis.

### 4.5. Albumin Endocytosis

Primary TECs were cultured in 35-mm glass-bottomed dishes to ~90% confluence over six days. The cells were incubated with FITC-BSA (100 µg/mL) in serum-free medium at 37 °C for 1–3 h. In another set of experiment, the cells were pretreated with DMSO or simvastatin (10 µM) for 2 h, and then exposed to FITC-BSA for another 3 h. Afterwards, the cells were washed with ice-cold PBS, then fixed in 4% formaldehyde for 15 min. Images were captured by a Leica confocal microscope (Wetzlar, Germany).

### 4.6. Immunofluorescence Staining

To examine the distribution of endogenous albumin and MMP-9 in renal tubules, dual labeling was performed by incubating 5-µm-thick cryostat kidney sections with a mixture of two primary antibodies overnight: sheep anti-rat albumin (1:100; A110-134A, Bethyl Laboratories, Inc., Montgomery, TX, USA) with rabbit anti-aquaporin-1 (1:100; EMD Millipore, Billerica, MA, USA), or rabbit anti-MMP-9 (1:100; Abcam, Cambridge, MA, USA) with goat anti-rat KIM-1 (1:100; R&D Systems, Minneapolis, MN, USA). The secondary antibodies were FITC-conjugated donkey anti sheep/rabbit IgG (1:200) or Rhodamine-labeled donkey anti goat/rabbit (1:200) from Jackson ImmunoResearch Laboratories (West Grove, PA, USA). Negative control was done by replacing the primary antibodies with nonimmune IgG. No specific staining was observed in negative controls.

### 4.7. Gelatin Zymography

Rat urine and culture supernatants from primary TECs were used to evaluate the gelatinolytic activity of MMPs as described previously [16,35,36]. The gels were stained with Brilliant Blue R Staining Solution (Sigma Aldrich Inc.) and scanned using white light transillumination. ImageJ 1.46r software (NIH, Washington, DC, USA) was used to determine the relative density of each gelatinolytic band.

### 4.8. Immunoblot Analysis

For Western blot analysis, rat urine, cell culture supernatants, and cell lysates were separated by 10% SDS-PAGE and transferred to nitrocellulose membrane. The membranes were routinely stained by Ponceau S (Sigma Aldrich Inc.). The primary antibodies were rabbit anti-rat MMP-9 (1:3000), rabbit anti-megalin (1:1000, Abcam), mouse anti-TGF-β1 (1:250, Santa Cruz), and mouse anti β-actin or α–tubulin (1:5000; Sigma Aldrich Inc.). HRP-conjugated secondary antibodies were goat anti rabbit (1:5000), mouse anti-goat (1:5000) and goat anti-mouse (1:5000) purchased from Santa Cruz Biotechnology (Santa Cruz, CA, USA). Detection was accomplished using enhanced chemiluminescence Western blotting (ECL, GE Healthcare, Piscataway, NJ, USA). For urine and cell culture supernatants, Ponceau red staining was used for loading control. Either β-actin or α-tubulin was used as an internal control for cell lysates. Relative band intensity was measured densitometrically using ImageJ software.

### 4.9. Statistical Analysis

All results are expressed as mean ± SEM. Statistical significance (considered when *p* < 0.05) was determined by either Student’s *t* test for comparison between two groups, or by one-way ANOVA with a Newman–Keuls post hoc test for comparisons among multiple groups.

## 5. Conclusions

Our data show increased tubular expression of MMP-9 in activated proximal tubules in diabetic rat kidneys. Notably, an upregulation of tubular MMP-9 was associated with protein overload in ZDF rat tubules, which could be prevented by anti-proteinuric treatment. Albumin exposure stimulated MMP-9 release from cultured TECs through an activation of ERK1/2 MAPK signaling pathway. Our results also indicate that internalization of albumin is required for albumin-induced MMP-9 production by TECs. Therefore, emphasis should be placed on reducing glomerular leakage of albumin and albumin uptake by proximal TECs from the tubular lumen in chronic kidney disease.

## Figures and Tables

**Table 1:** General metabolic parameters of vehicle-treated Zucker diabetic fatty (ZDF) and rosiglitazone (Ros)-treated ZDF rats.

Header	ZDF (*n* = 8)	Ros-ZDF (*n* = 7)	*p* Value
Food intake (g)	29 ± 2	33 ± 1	0.1529
Water intake (mL)	57 ± 7	42 ± 3	0.0635
Urine volume (mL/day)	34 ± 5	15 ± 1	0.0017
Urinary protein (mg/day)	326 ± 34	149 ± 20	0.0005
Protein/creatinine	36 ± 3	17 ± 2	0.0002
Cholesterol (mg/dL)	168 ± 11	159 ± 15	0.6566
Triglyceride (mg/dL)	462 ± 80	30 ± 6	0.0002
Body weight (g)	407 ± 22	654 ± 16	0.0001
Pancreas weight (g)	0.69 ± 0.04	0.87 ± 0.05	0.0153
Pancreas/body weight (‰)	1.77 ± 0.14	1.28 ± 0.09	0.0154
Kidney weight (g)	1.58 ± 0.07	1.46 ± 0.03	0.1408
Kidney/body weight (‰)	3.94 ± 0.26	2.15 ± 0.04	0.0001
Heart weight (g)	1.07 ± 0.04	1.60 ± 0.04	0.0001
Heart/body weight (‰)	2.66 ± 0.09	2.35 ± 0.09	0.034

## Abbreviations

ERK	Extracellular signal-regulated kinase
MMP	Matrix metalloproteinase
TECs	Tubule epithelial cells
ZDF	Zucker diabetic fatty
ZL	Zucker lean
RSA	Rat serum albumin
BSA	Bovine serum albumin
DN	Diabetic nephropathy
RANTES	Regulated on activation, normal T cell expressed and secreted
MCP-1	Monocyte chemoattractant protein-1
TGF-β	Transforming growth factor-β
ECM	Extracellular matrix
EMT	Epithelial-mesenchymal transition
KIM-1	Kidney injury molecule-1
